# Grenzen und Fortschritte indikatorengestützter Politik am Beispiel der Corona-Pandemie: Heinz-Grohmann-Vorlesung 2020/21

**DOI:** 10.1007/s11943-022-00314-6

**Published:** 2022-11-21

**Authors:** Gert G. Wagner

**Affiliations:** 1 Max Planck Fellow, MPI für Bildungsforschung, Berlin, Deutschland; 2 Sozio-oekonomisches Panel (SOEP), Berlin, Deutschland; 3 Harding-Zentrum für Risikokompetenz, Potsdam, Deutschland; 4 Bundesinstitut für Bevölkerungsforschung (BiB), Wiesbaden, Deutschland

**Keywords:** Indikatoren, Instrumente, Risikokommunikation, Indicators, Instruments, Risk communication, C80, I30, I31, P11, Z18

## Abstract

Indikatoren sollen der Steuerung von (sozialen) Prozessen dienen. Sie beschreiben jedoch die Realität in der Regel nur deskriptiv und unvermeidlich mit mehr oder weniger großen und systematischen Messfehlern behaftet. Insofern ist es im Allgemeinen alles andere als einfach mit Hilfe von Indikatoren zu steuern; insbesondere dann, wenn für Problembereiche (fehlerbehaftete) Zielwerte vorgegeben werden, für deren Erreichung die Indikatoren keine kausalen Pfade aufzeigen. Am Beispiel der Corona-Pandemie werden statistisch-methodische Probleme von (sozialen) Indikatoren aufgezeigt und auf ein zentrales Defizit der vorhandenen bzw. rasch entwickelten Pandemie-Indikatoren hingewiesen: fehlende Indikatoren für Instrumente der Risikokommunikation. Als eine positive Nebenwirkung der Pandemie wird die verbesserte „statistical literacy“ hervorgehoben und diskutiert.

## Abstract (en)

Indicators are intended to serve the control of (social) processes. However, they usually describe reality only descriptively and are inevitably subject to more or less large and systematic measurement errors. In this respect, it is generally anything but easy to control with the help of indicators; especially when (error-laden) target values are specified for problem areas, for the achievement of which the indicators do not show any causal paths. Using the Corona pandemic as an example, statistical-methodological problems of (social) indicators are highlighted and a central deficit of existing or rapidly developed pandemic indicators is pointed out: lack of indicators for risk communication instruments. Improved “statistical literacy” is highlighted and discussed as a positive side effect of the pandemic.

Die Heinz-Grohmann-Vorlesung im September 2021 war für den Autor dieses Beitrags ein willkommener Anlass auf seinen wissenschaftlichen Werdegang zurückzublicken, der unmittelbar mit dem Hochschullehrer Heinz Grohmann verbunden ist, da der Autor im Grund- und Hauptstudium der Volkswirtschaftslehre in Frankfurt am Main von Heinz Grohmann Statistik gelehrt bekommen und mit ihm im Sonderforschungsbereich 3 zusammengearbeitet hat, wo soziale Indikatoren und ihre Simulation eine zentrale Rolle spielten (vgl. Krämer 2022). Dieser persönliche Hintergrund ist natürlich zweischneidig: einerseits kennt man sich in einem Thema, das einem ein ganzes Berufsleben lang begleitet, gut aus (zumindest besser als in anderen Gebieten), andererseits sind viele persönliche Erinnerungen mit Spezialthemen und den Personen, die diese bearbeitet haben, verbunden. Und diese Erinnerungen führen unvermeidbar zu einem sehr subjektiven Blick auf die Entwicklung; und damit verbunden wird der einen oder dem anderen Kolleg:in unrecht getan. Der Autor des vorliegenden Beitrags konnte es trotzdem nicht lassen, sich seiner eigenen Vergangenheit zu stellen – zumal (soziale) Indikatoren während der Corona-Pandemie eine enorme Relevanz gewonnen haben; und nicht nur in Form der die Pandemie beschreibenden Kennziffern, sondern einige Kennziffern wurden explizit zu (gesetzlichen) Zielvorgaben für die Pandemie-Bekämpfung gemacht (etwa „die Inzidenz“). Fast zwei Jahre lang hat die Politik anhand verschiedener Indikatoren gesteuert bzw. zu steuern versucht. Es ist offenbar intellektuell reizvoll und für künftige Pandemien vielleicht ein wenig nützlich die (Irr)Relevanz der Corona-Indikatoren näher zu betrachten. Dies geschieht nicht aus der Warte des Septembers 2021 (als die Vorlesung gehalten wurde), sondern unvermeidbar aus der Warte zum Zeitpunkt der ersten Überarbeitung des Vortragsmanuskripts (Anfang April 2022); wodurch einige Analysen nochmals geschärft wurden. Deswegen wurde der vorliegende Beitrag mit dem Titel „Grenzen und Fortschritte indikatorengestützter Politik am Beispiel der Corona-Pandemie“ überschrieben – und nicht mit dem Titel der Vorlesung im Jahr 2021: „Zur Bedeutung von (sozialen) Indikatoren für die Steuerung von (sozialen) Prozessen am Beispiel der Corona-Politik.“

## Um was geht es?

Moderne Zivilisationen sind ohne quantifizierte bzw. statistische Indikatoren nicht vorstellbar. Auch wenn sie nicht immer so genannt werden, sind Kennziffern zur Beschreibung des Status Quo entscheidend für viele Entscheidungen. Bereits die ersten Zivilisationen, so etwa im Zweistromland, waren ohne Buchhaltung nicht denkbar, und der moderne Kapitalismus ist auf doppelte Buchführung angewiesen. Diese Beispiele machen bereits deutlich, dass gute Indikatoren zwar von zentraler Bedeutung sind, aber deswegen keineswegs problemlos und ein für alle Male ausgereift. Nach wie vor ist eine gute Buchhaltung Gegenstand von Forschung, dazu gehört wie Indikatoren möglichst angemessen (adäquat, wie Heinz Grohmann sagen würde) definiert und gemessen werden und wie sie gegen Manipulationen und Betrug geschützt werden können, die das Bild der Wirklichkeit, das die Indikatoren vermitteln sollen, verzerren und ggf. wertlos machen. Und die Geschichte der Buchhaltung und Indikatorik zeigt auch, dass in einer dynamischen Welt quantitative Beschreibungen, die naturgemäß bestenfalls den „aktuellen Rand“ zeigen, oft aber auch nur die jüngere Vergangenheit, nicht ausreichen, um auch in Zukunft erfolgreich zu sein, also die Ziele zu erreichen, die man mit Hilfe der Indikatoren erreichen will. Insofern macht schon dieser kurze Blick auf die Erfahrungen, die mit Indikatoren im Bereich der Buchhaltung gemacht werden, deutlich, dass es vermessen wäre zu erwarten, dass es perfekte Indikatoren gäbe, die uns hätte helfen können besser durch die Corona-Pandemie zu kommen als das der Fall war.

Um es zuzuspitzen: die Erwartungen, die einige Statistiker und Epidemiologen geweckt haben (vgl. für einen – nicht repräsentativen – Überblick z. B. Breitling und Dittrich 2022), dass mit Hilfe besserer Epidemiologie bzw. Pandemie-Statistiken wir besser durch die Corona-Pandemie gekommen wären, waren überzogen – zumal die offenbar wichtigsten Indikatoren gar nicht im Mittelpunkt der amtlichen und akademischen Statistik stehen; nämlich Indikatoren, die zeigen welche Kommunikations*maßnahmen* die Menschen am ehesten überzeugen sich in einer Pandemie vernünftig zu verhalten (vgl. z. B. Schöps 2022 und die unten stehenden Ausführungen).

Es war sicherlich richtig während der Pandemie (mit einer gewissen Hektik) möglichst viel Daten zu erheben, damit sie für Analysen vorliegen. Es war aber falsch zu glauben, diese Daten würden bei Entscheidungen viel helfen. Faktisch – so wage ich zu behaupten – wurde ohnehin im Wesentlichen anhand der Belastung des Gesundheitswesens gesteuert bzw. zu steuern versucht (vgl. z. B. Kubicki 2022): „Die Überlastung des Gesundheitssystems zu verhindern, war immer das oberste staatliche Ziel aller Maßnahmen.“ Und das war vielleicht gar nicht unvernünftig (vgl. z. B. Hertwig und Wagner 2020). Alle anderen Indikatoren, allen voran „die Inzidenz“, waren nur Hilfsindikatoren, von denen erwartet wurde, dass sie kommunikativ effektiv wirken, also uns alle davon überzeugen uns vernünftig zu verhalten. Diese Wirkung gelang im Laufe der Zeit unterschiedlich gut – mit rationaler Steuerung anhand gut gemessener statistischer Indikatoren hatte das allerdings wenig zu tun, da – wie gesagt – Indikatoren fehlten, die die Wirksamkeit kommunikativer Maßnahmen prognostizieren konnten.

Es sei schon hier gesagt: ein wesentlicher Fortschritt, zu dem die Pandemie ungewollt geführt hat, ist der Beweis dafür, dass die Öffentlichkeit bereit ist methodische Überlegungen, die für die Interpretation von statistischen Ergebnissen wichtig sind, zu rezipieren (z. B. Konfidenzbänder), wenn dies – was bei der Pandemie der Fall war – als für das eigene Leben als relevant angesehen wird.

Im Folgenden wird zuerst kurz die Geschichte der Entwicklung „sozialer Indikatoren“ in Deutschland skizziert (Abschn. 2), um dann in Abschn. 3 ausgewählte Probleme der in der Pandemie benutzten Indikatoren zu diskutieren und schließlich in Abschn. 4 aus dem Umgang mit Statistiken während der Pandemie einige allgemeine Schlussfolgerungen zu ziehen.

## Ganz kurzer Blick auf den historisch-politischen Hintergrund sozialer Indikatoren (und von Mikrosimulation)

Mit dem in den 60er-Jahren gestarteten „Social Indicator Movement“ wurde versucht die Idee einer rationalen Steuerung der Gesellschaft tatsächlich umzusetzen (vgl. z. B. Noll 2016 sowie Glatzer und Wagner 2014). Der Zustand einer Gesellschaft solle mit (sozialen) Indikatoren beschrieben werden, deren Werte zumindest zum Teil auch zu politischen Zielen gemacht werden sollten; also Werte, die die Politik realisieren solle. Im politischen Prozess würde Erfolg an der Erreichung dieser Zielwerte gemessen werden, aber die Zielwerte könnten und sollten ggf. auch verändert werden. Es solle also einen Rückkopplungsprozess geben (Fox-Tinbergen-Land-Schema), der mehr Rationalität in die Politik bringen sollte (vgl. z. B. Krupp und Zapf 1977; Kade et al. 1972 sowie Fehl et al. 1972, zusammenfassend und rückblickend Mayer 2018).

Die Idee mit Hilfe von quantitativen Indikatoren rational(er) politisch zu steuern gewann nach der Jahrtausendwende (wieder) eine gewisse politische Attraktivität. Im Auftrag des französischen Präsidenten Sarkozy haben u. a. die Nobelpreisträger Arrow, Heckman, Kahneman, Sen und Stiglitz, zusammen einer größeren Gruppe von Experten, ein Konzept vorgelegt (Stiglitz et al. 2009), das politisch allerdings zu nichts geführt hat. Dies könnte u. a. auch daran liegen, dass die Sozialindikatoren-Bewegung niemals versucht hat zu analysieren (und ggf. Indikatoren dafür zu entwickeln) wie eine effektive Kommunikation der in Indikatoren enthaltenen Informationen in die Politik hinein erfolgen kann. Vielmehr wurde schlicht angenommen, dass die Politik für Indikatoren dankbar sei. Der seinerzeitige „Wirtschaftsweise“ Christoph Schmidt (2018, S. 124) stellte dazu fest: „Die vergleichsweise junge Berichterstattung ‚jenseits des BIP‘ [hat] ihre Rolle letztlich noch nicht ganz gefunden.“ Dies gilt nach Überzeugung des Autors auch für entsprechende internationale Bemühungen.

Im Jahr 2015 hat die UNO ein System von Zielen, von denen etliche quantifiziert sind (aber keineswegs alle), vorgelegt: die Sustainable Development Goals (SDG) (https://sdgs.un.org/goals). Diese Ziele werden gerne in politischen Auseinandersetzungen zitiert bzw. angestrebt oder abgelehnt. Von *effektiver *politischer Bedeutung, d. h. tatsächlicher Handlungsrelevanz dieser Indikatoren, kann man – nach Ansicht des Autors – aber nicht sprechen (vgl. dazu auch Tiemann und Wagner 2013).

Die (Natur)Wissenschaftlerin Angela Merkel hat – im Nachgang zu einer einschlägigen Enquete-Kommission (vgl. Deutscher Bundestag 2013) – mit dem Regierungsprojekt „Gut leben in Deutschland“ versucht quantitative Zielwerte in den politischen Prozess zu bringen (https://www.gut-leben-in-deutschland.de/, https://buergerdialog.gut-leben-in-deutschland.de/DE/Ueber/Hintergrund/_node.html). Im politischen Alltag (und auch im öffentlichen Diskurs) hat das entwickelte Indikatorensystem keinerlei Bedeutung erlangt (darauf wird am Ende von Abschn. 3 unten näher eingegangen).

Es gibt freilich auch Indikatoren, die als Zielwerte auf höchster Ebene gesetzlich festgeschrieben sind. In Deutschland auf die Spitze getrieben wurde das Konzept der politischen Steuerung mittels eines Indikators mit der grundgesetzlichen Festschreibung der „Schuldenbremse“ (die niedrige Höchstwerte für die Netto-Neuverschuldung des Staates vorsieht). Allerdings wird dabei nicht berücksichtigt, dass es neben einer in Grenzen zu haltenden Staatsverschuldung vernünftigerweise weitere politisch-staatliche Ziele gibt und nicht alle simultan erfüllbar sind. Die Verabsolutierung des öffentlichen Schuldenstands ließ von vorne herein Umgehungsstrategien erwarten, die im Zuge der Corona- und Verteidigungs-Politik auch prompt eine Rolle spielen, nämlich Schattenhaushalte. In der Literatur wird die Umgehung einzelner politischer Ziele unter dem Stichwort „Goodhart’s Law“ diskutiert (vgl. z. B. Wagner 2021)^1^. Angemerkt sei: Die Väter des „Stabilitäts- und Wachstumsgesetzes“ waren in den 60er-Jahren klüger als die Große Koalition, die die Schuldenbremse ins Grundgesetz schrieb: die konkurrierenden wirtschaftspolitischen Ziele im Stabilitäts- und Wachstumsgesetz (vgl. z. B. https://www.bpb.de/kurz-knapp/lexika/lexikon-der-wirtschaft/20711/stabilitaetsgesetz/) sind nicht quantifiziert, sondern sie werden mit *unbestimmten* Rechtsbegriffen beschrieben: so insbesondere „hoher“ Beschäftigungsstand sowie „stetiges und angemessenes“ Wirtschaftswachstum. Und was man sich unter Preisniveaustabilität und einem außenwirtschaftlichen Gleichgewicht vorstellen sollte, war immer wieder Gegenstand wissenschaftlicher Kontroversen.

Verfechter sozialer Indikatoren könnten nun argumentieren, dass die Schuldenbremse kein umfassendes Zielsystem sei, sondern fälschlicherweise ein einziges Ziel verabsolutiert wird. Das stimmt, aber auf der anderen Seite haben die umfassenderen Indikatoren-Systeme zumindest bislang kein politisches Gewicht gewonnen. Die Sarkozy/Stiglitz-Indikatoren sind ein Beispiel (die meines Erachtens weniger am neoliberalen Zeitgeist als an ihrer Komplexität scheiterten). Oder das öffentlich kaum beachtete und nahezu unbekannte – oben bereits angesprochene – Indikatorensystem „Gut leben in Deutschland“ (vgl. z. B. Wagner 2016). Für dessen Wirkungslosigkeit sind in erster Linie politische und organisatorische Gründe verantwortlich, nämlich die Konkurrenz verschiedener Politikbereiche und der verschiedenen Ministerien, aber auch ganz banale Messprobleme, auf die am Beispiel der Corona-Indikatoren in Abschn. 3 beispielhaft im Detail eingegangen wird.

Hier sei noch darauf hingewiesen, dass Indikatoren, die der Steuerung von (sozialen) Prozessen dienen, nicht nur unvermeidlich mit Messfehlern behaftet sind (was den öffentlichen Diskurs verwirren kann), sondern die Indikatoren in der Regel die Realität bzw. enge Ausschnitte der Realität auch nur deskriptiv beschreiben (und man dadurch nicht auch weiß, wie man die Realität beeinflussen kann; vgl. etwa Wagner 2002). Insofern ist es im Allgemeinen alles andere als einfach mit Hilfe von Indikatoren zu steuern; insbesondere dann, wenn für selektiv ausgewählte Problembereiche Zielwerte vorgegeben werden, für deren Erreichung kausale Pfade nicht mit Hilfe von Indikatoren beschrieben werden (können).

Ein zentrales Problem besteht darin, dass statistische Indikatoren sozusagen immer veraltet sind, da sie ja nur für die Vergangenheit gemessen werden können. Der „aktuelle Rand“ wird in der Regel nicht erreicht, sondern bestenfalls simuliert^2^ – und das nach Ansicht des Autors viel zu wenig (zu „Nowcasts“ unten mehr; vgl. auch De Nicola 2022). Der Hinweis eines der Gutachter, die den Entwurf des vorliegenden Aufsatzes begutachtet haben, dass beispielsweise Arbeitsmarkt-Indikatoren monatlich veröffentlicht werden, also am aktuellen Rand, und subjektive Konjunktur-Indikatoren eine Vorausschau auf die kommenden Monate geben, ist richtig, bestätigt aber nur die Ausnahme von der Regel (und das Beispiel macht auch deutlich, dass auch ganz aktuelle Indikatoren keinerlei sichere Prognosen zulassen).

Interessanterweise hatte sich die Sozialindikatoren-Bewegung in Deutschland bereits in den 70er-Jahren mit Entwicklern von Simulationssystemen zusammen getan. Der Pionier der Sozialindikatorenbewegung in Deutschland, der Soziologe Wolfgang Zapf, und der Ökonom Hans-Jürgen Krupp haben zusammengearbeitet (anfänglich in Frankfurt am Main, später in Zusammenarbeit von Frankfurt und Mannheim). Name und Acronym des hoffungsvollen SPES-Projektes (DFG finanziert) waren Programm: Sozialpolitisches Entscheidungs- und Indikatorensystem (vgl. auch Krämer 2022). Hinter dem „Entscheidungssystem“ standen Simulationsmodelle; zuerst makroökonomische Zeitreihenmodelle und danach *–* im SPES-Nachfolgeprojekt: dem Sonderforschungsbereich 3 *–* ein Mikrosimulationsmodell, das auf der amtlichen Einkommens- und Verbrauchs-Stichprobe (EVS) von Personen und Haushalten beruhte (vgl. dazu Hauser et al. 1994). In der Terminologie von modernen Simulationssystemen würde man heute von einem „Agentenmodell“ reden; wobei die Interaktion beim Sfb 3‑Mikrosimulationsmodell überschaubar war: sie beschränkte sich auf das Heiraten von zwei Agenten (die damals noch strikt Paare von Datensätzen je einer Frau und eines Mannes waren) (vgl. Galler und Wagner 1986). Bei der Modellierung einer Pandemie (oder zum Beispiel des Autoverkehrs) spielen naturgemäß viel mehr Interaktionen zwischen einer Vielzahl von „Agenten“ eine Rolle.

Das im Sonderforschungsbereich entwickelte dynamische Mikrosimulationsmodell hat nie eine politische Bedeutung erlangt. Nun kann man argumentieren, dass es zu früh kam, da zwar die erforderliche Computerleistung in den 80er-Jahren vorhanden war, aber nicht die Datenbasis, die es erlaubt hätte empirisch robuste und prognosefähige Verhaltenshypothesen statistisch zu schätzen und in das Modell einzubauen. Ob diese Einschätzung wirklich zutrifft spielt hier keine wesentliche Rolle, vielmehr ist es für die vorliegende Diskussion ausschlaggebend, dass alle bekannten sozialen Indikatorensysteme gar nicht erst versuchen den aktuellen Rand (oder gar die nahe Zukunft) zu simulieren, sondern alle Indikatoren nur rückwärts schauen. Dies ist erstaunlich, da es inzwischen eine große Zahl von (Mikro)Simulationssystemen gibt, die die kurz- bis langfristigen Wirkungen von Reformen abschätzen, etwa im Bereich der Steuer- und Transferpolitik und insbesondere im Bereich Klima(wandel) (vgl. z. B. https://www.ifo.de/node/67074 und https://www.umweltbundesamt.de/themen/klima-energie/klimafolgen-anpassung/folgen-des-klimawandels/klimamodelle-szenarien#was-sind-treibhausgasszenarien-und-wofur-werden-sie-genutzt). Es wäre also möglich den aktuellen Rand mit „Nowcasts“ zu schätzen (vgl. Wagner 2022, Abschn. 5.2) – wie dies für die Entwicklung der Corona-Pandemie geschieht (siehe Abschn. 3 unten).

## Probleme von statistischen Indikatoren am Beispiel der Corona-Pandemie

Statistische Indikatoren sind unvermeidbar mit Messfehlern behaftet und geben in der Regel nicht den wirklich aktuellen Rand der Wirklichkeit wieder und allein deswegen fällt Steuerung anhand von Indikatoren schwer. Bei der Pandemie wurde dies besonders deutlich.

Bei der Feststellung des aktuellen Pandemie-Geschehens gab es – nicht nur – in Deutschland ganz praktische Messprobleme, die auch durch nicht-digitale Meldewege verursacht wurden. Doch selbst digitale Meldungen in Echtzeit an ein zentrales Register hätten nicht verhindert, dass an Wochenenden weniger getestet wird als an Wochentagen (sofern auf diese nicht ein Feiertag fällt). Dadurch hat sich ein „saisonales“ Muster bei den Meldungen nach Wochentagen ergeben, für das in der Kommunikation und der öffentlichen Debatte recht zügig eine einfache und gute Lösung gefunden wurde: der Vergleich der Meldungen für dieselben Wochentage bzw. der Vergleich der Meldungen für komplette Wochen.

Dieser Lernprozess ist das erste Beispiel für meine These, dass die Öffentlichkeit sehr wohl in der Lage ist gewissermaßen die „methodischen Fußnoten“ von Statistiken wahrzunehmen. Voraussetzung ist freilich, dass die methodischen Probleme als für die Lebenswirklichkeit relevant angesehen werden. Dann sind auch Journalistinnen und Journalisten bereit, sich mit methodischen Problemen zu beschäftigen, und ihre Reflektionen stoßen auf eine interessierte Leser‑, Zuhörer- und Zuschauerschaft. Höhle (2022, S. 385) weist zu Recht darauf hin, dass insbesondere auch auf Daten und ihre Interpretation spezialisierte Datenjournalisten in der Pandemie an Bedeutung gewonnen haben – was auch für die akademische Statistik eine erfreuliche Entwicklung ist.

Da die täglich gemeldeten Infektionen jedoch wegen der Inkubationszeit der Corona-Infektion nicht das Infektionsgeschehen am Meldungs-Tag wiedergeben, wurde für die zusammenfassende Beschreibung der Infektionsdynamik mit Hilfe des R‑Wertes (anfänglich war es die Verdopplungszeit)^3^ vom RKI ein „Nowcast“ entwickelt (vgl. an der Heiden und Hamouda 2020), der bis zum wirklich aktuellen Rand reicht, aber annahmebehaftet ist und deswegen wird er mit einem Konfidenzintervall ausgewiesen. Auch hier kann man lernen: wenn das Publikum diese methodische Feinheit als wichtig erachtet, ist es bereit sich damit zu beschäftigen und Viele werden die Grenzen der Aussagefähigkeit des aktuellen R‑Wertes auch erkannt haben. Deswegen liegt die Frage nahe: warum wird eigentlich nicht für die Verteilungsstatistiken mit Nowcasts gearbeitet, sondern nach wie vor auf Basis komplizierter Stichproben mit bis zu zwei Jahren veralteten Daten (vgl. Wagner 2022, Abschn. 5.2).

Trotz der raschen Verbesserungen der statistischen Beschreibung der Pandemie blieb ein Problem ungelöst: die Dunkelziffer asymptomatischer Infektionen konnte zu keinem Zeitpunkt auf Basis aktueller Informationen aufgehellt werden. Da für die Erfassung unbemerkt erfolgter Infektionen spezielle Erhebungen notwendig sind, gelang es auch in keinem Land der Welt den wirklich aktuellen Rand des vollen Infektionsgeschehens statistisch zu erfassen, sondern mit Hilfe von mehr oder weniger veralteten Stichproben wurde die Dunkelziffer mit mindestens einigen Tagen bis etwa zwei Wochen Time-lag abgeschätzt. Da bei Stichproben ein Zufallsfehler unvermeidbar ist, sind die so gewonnenen Inzidenzzahlen unvermeidlich nur Schätzungen.

Ob in irgendeinem Land so die „wirkliche“ Inzidenz gemessen wurde, ist zweifelhaft, da es für bestimmte Personengruppen (etwa Tagelöhnern und anderen prekär Beschäftigten) einen Anreiz gibt, sich nicht testen zu lassen, wenn ein positives Testergebnis mit der Meldung zu einer Behörde und damit mit einer Quarantäne und einem Einkommensausfall verbunden ist (vgl. Rendtel et al. 2021, Fußnote 61).

Interessanterweise wird inzwischen eine höchst indirekte Messung des Pandemiegeschehens für wichtig (und richtig) gehalten, obwohl diese wenig anschaulich ist und ggf. nicht einfach zu kommunizieren: die Messung der Virusbelastung des Abwassers. Der ExpertInnenrat der Bundesregierung stellt perspektivisch ausdrücklich fest (2022b, S. 12), dass künftig die „Identifikation von lokalen Ausbrüchen oder erhöhtem Infektionsgeschehen durch die Etablierung des Abwassermonitorings in Bezug auf SARS-CoV-2“ erfolgen sollte (vgl. auch Wagenmakers 2022; Schumann et al. 2022).

Es stellt sich in der Tat die Frage, ob die Kenntnis des vollen bzw. wahren Infektionsgeschehens überhaupt notwendig ist, wenn die Inzidenz nur ein Hilfsindikator für die Steuerung der Pandemie ist – faktisch aber anhand der Belastung des Gesundheitssystems gesteuert wird bzw. versucht wird zu steuern (vgl. Kubicki 2022; Berndt 2022). Dem folgt seit Anfang 2022 auch das RKI (2022, S. 4) explizit: „RKI: Zudem steht für die Lagebewertung in der aktuellen Situation der Pandemie nicht die Erfassung aller Infektionen durch SARS-CoV‑2, sondern die Entwicklung der Anzahl und Schwere der Erkrankungen im Vordergrund.“

Grundsätzlich stellt sich die Frage was beschreibende Indikatoren für (Politik)Maßnahmen nutzen, wenn sie bestenfalls den aktuellen Rand wiedergeben, aber unklar ist wie Maßnahmen aussehen können bzw. müssen, die die Realität so verändern, dass der gemessene Indikator sich in die gewünschte Richtung bewegt. Also bei der Pandemie in Richtung weniger schwerer oder gar tödlicher Infektionen. Oder zum Beispiel im Hinblick auf den Klimawandel in Richtung weniger CO_2_-Ausstoß. Um in die Zukunft blicken zu können, was ja die Absicht ist, die hinter politisch relevanten Indikatoren steht, braucht man auch Indikatoren für die Abschätzung der Wirkung von (Politik)Instrumenten. Derartige Indikatoren sind aber von der Natur der Sache nach nicht durch deskriptive statistische Erhebungen zu gewinnen, sondern nur durch Simulationsstudien. Und bei solchen spielt wiederum der Natur der Sache nach Unsicherheit eine Rolle. Und bei beiden genannten Themen – Pandemie und CO_2_-Ausstoß – geht es letztlich darum wie man die Menschen zu vernünftigem (gewünschten) Verhalten bringt. Wie schwierig dies ist zeigt die Erderwärmung: es gibt immer wieder Streit darüber, welche Instrumente bzw. Politikmaßnahmen geeignet sind den globalen Temperaturanstieg zu begrenzen. Dabei geht es durchaus auch um ideologische Auseinandersetzungen, aber im Kern doch darum, dass wir eben nicht genau wissen was die besten Maßnahmen sind.

Das Problem reiner Deskription spricht auch der Volksmund aus, wenn er feststellt: „Vom Wiegen wird die Sau nicht fett“ (vgl. Wagner 2021). Gemeint ist: wenn ich nicht weiß wie ich ein Schwein mäste, nutzt auch ständiges Wiegen nichts. Es ist aber noch schlimmer: wenn ein gewünschtes Ziel, etwa der Preis, den ein Viehzüchter für ein Schlachtschwein erhält, vom Gewicht und damit vom Wiegen abhängt, dann besteht ein Anreiz das Schwein vor dem Verkauf – und dem Wiegen – möglichst billig mit Wasser anzufüllen, um einen höheren Preis zu erzielen.

Immerhin: Soweit man das überblicken kann hat der Hilfs-Indikator „Inzidenz“ in Deutschland nicht dazu geführt, dass versucht wurde durch weniger Testen die Inzidenz scheinbar zu senken (und selbst Donald Trump, der laut über diesen Weg nachgedacht hatte, hat das nicht getan).

Der Hilfs-Indikator „Inzidenz“, der die ersten eineinhalb Jahre der Pandemie eine zentrale Rolle für den politisch-administrativen und öffentlichen Umgang mit der Pandemie spielte, hatte auch ohne Manipulationsversuche recht offensichtlich eine negative Seite: niedrige Inzidenzen haben zu Leichtsinn geführt – sowohl kollektiv wie individuell. Das „Wiegen“ der Pandemie hat also zeitweise dazu geführt, dass die Vorsicht leichter wurde und nicht schwerer. Klüger wäre es gewesen auf Prognosen, die es ja rasch gab, zu hören. Das geschah – wie berichtet wurde – durchaus, etwa bei Kanzlerin Merkel und ihrem Kanzleramtschef, aber beide konnten sich oft nicht gegen die Ministerpräsidenten, die mit Verweis auf niedrige gemessene Inzidenzen Lockerungen wollten, durchsetzen. Dadurch hat sich aus meiner Sicht – ohne dass ich freilich dafür einen empirisch belastbaren Beleg hätte – seit 2021 das Problem ergeben, dass leichtsinnig gewordene Geimpfte (die ja das Virus übertragen konnten) ungewollt viele Nicht-Geimpfte infiziert haben.

Hinzu kam: wenn die Überschreitung von Grenzwerten, die politische Aktionen auslösen sollten (wie die z. B. in Berlin die Corona-Ampel für die Inzidenz) wochen- oder am Ende monatelang zu keinerlei erkennbarer politischer Aktion führt, wirken Indikatoren im Zweifel kontraproduktiv, da sie nicht mehr ernst genommen werden. Dann nutzt es auch nichts, wenn die Pandemie zu einem kollektiven Nachhilfekurs in Sachen Statistik und Verständnis für Wissenschaft und ihre Methode geführt hat.^4^ Das war zweifelsohne der Fall: die Öffentlichkeit hat viel über Mess- und Stichprobenfehler bei Statistiken gelernt, da etliche Medien – insbesondere im Internet – detailliert Pandemie-Indikatoren laufend veröffentlicht und mit viel Hintergrundwissen versehen haben (vgl. z. B. https://www.rbb24.de/panorama/thema/2020/coronavirus/service/faelle-berlin-brandenburg-verdopplungszeit-fallzahlen-entwicklung.html sowie Peeples 2022). Pasternack und Beer (2022, S. 9 und S. 70 f) sprechen sogar – zu recht – von einem „Schnellkurs in Wissenschaftstheorie“, da ja immer wieder die Aussagefähigkeit von Studien öffentlich diskutiert wurde (bis hin zu Problemen des Peer-Review).

Was auffällt: die Kommunikationswissenschaft wurde nach allem was man weiß von der Politik nicht systematisch gehört – aber die Kommunikationswissenschaft hat sich auch kaum öffentlich zu Wort gemeldet. Rein akademische Veröffentlichungen sind zwar relativ rasch gekommen (vgl. z. B. Wegwarth et al. 2020), aber die Wissenschaft von der Risikokommunikation war erstaunlich schweigsam und dies obwohl zum Beispiel das RKI ausdrücklich sich im Bereich Risikokommunikation verstärkt hat.

Erst sehr spät hat die Politik Wissenschaftlerinnen und Wissenschaftler im Bereich Gesundheits- und Risikokommunikation systematisch einbezogen (zur Kritik vgl. z. B. Ludwig 2021, S. 10), nämlich in den im Dezember 2021 berufenen „ExpertInnenrat der Bundesregierung zu COVID-19“, dessen 5. Stellungnahme sich Ende Januar 2022 mit der „Notwendigkeit evidenzbasierter Risiko- und Gesundheitskommunikation“ beschäftigte (ExpertInnenrat 2022a). Interessanterweise wird im einschlägigen Schwerpunktheft des „Allgemeinen Statistischen Archivs“ von Berger et al. (2022, S. 388f) inzwischen „Reporting und Communication“ als ein auch für Statistiker wichtiger Bereich herausgestellt.

Inzwischen liegen von hochrangigen Gremien zwei Empfehlungskataloge vor, die die Bedeutung der Risiko-Kommunikation ausdrücklich hervorheben: zum Ersten hat der ExpertInnenrat der Bundesregierung im Juni 2022 dies in seiner 11. Stellungnahme „Pandemievorbereitung auf Herbst/Winter 2022/23“ betont (ExpertInnenrat 2022b, S. 7)^5^, zum Zweiten eine von der renommierten Fachzeitschrift „The Lancet“ eingesetzte Kommission (Sachs et al. 2022). Die Kommission nennt 12-mal den Begriff „Communication“, so u. a. als „Key recommendation“ (S. 3) „Effective health communication strategies; active efforts to address public health disinformation on social media; and continuously updated evidence syntheses“ sowie (S. 43) die weltweite Notwendigkeit von „response capacity and communications expertise.“

## Fazit

Was lehrt uns die Corona-Pandemie im Hinblick auf die Nützlichkeit der politischen Steuerung mit Hilfe statistischer Indikatoren? Aus Sicht des Autors sind es zwei ganz unterschiedliche Lektionen. Und eine ist positiv.

Die negative Lektion ist nicht neu, sondern sie ist die zentrale Schwäche statistischer Indikatoren seit es diese gibt: die deskriptive Beschreibung der Welt hilft nur begrenzt weiter, wenn man die Welt gestalten will. Wenn die Statistik keine Indikatoren zur Verfügung stellt, die als Instrumente für die Veränderung der Welt geeignet sind, dann nutzen noch so komplexe Systeme „sozialer Indikatoren“ wenig – und sie können sogar negativ wirken. Ein „klassisches“ Beispiel ist ein Indikator, der sogar im deutschen Grundgesetz niedergeschrieben ist, die „Schuldenbremse.“ Diese sieht auf den ersten Blick äußerst rational aus (mit bis auf eine Nachkommastelle genauen Grenzwerten für die Staatsverschuldung), aber wegen der Konkurrenz mit anderen staatlichen bzw. politischen Zielen zur Umgehung geradezu auffordert (was in der Pandemie und bei der Änderung der deutschen Wehrpolitik unmittelbar beobachtbar ist).

Da die statistischen Indikatoren, die zur Beschreibung des Pandemiegeschehens benutzt wurden, keine Instrumentenvariablen umfassten (also Indikatoren, die die Instrumente beschreiben, die zur Erreichung von Zielen notwendig sind), sondern nur den Status Quo (unvermeidlich messfehlerbehaftet und mit einem gewissen Time-lag) beschrieben haben (Inzidenz, Hospitalisierung und Auslastung der Intensivstationen), haben sie nur begrenzt geholfen politisch zu steuern. Indikatoren, die die Wirksamkeit der Risikokommunikation gemessen hätten, fehlten völlig. Am Ende haben überschrittene Grenzwerte (rote Ampeln), die zu keiner erkennbaren politischen Aktion geführt haben, vielleicht mehr geschadet als genutzt? Mangels entsprechender Wirkungsforschung im Hinblick auf die Gesundheits- und Risikokommunikation wissen wir das allerdings nicht. Ausdrücklich sei angemerkt: zu Beginn der Pandemie war das Unwissen groß und die Wahl der Inzidenz als zentraler Indikator für die öffentliche Kommunikation war naheliegend. Umso interessanter ist die (unbeantwortete) Frage, warum es so lange dauerte bis die Politik offen zugab, dass faktisch die Belastung des Gesundheitswesens der zentrale Indikator für den Versuch der Steuerung des Pandemiegeschehens war.

Diese Situation unterstreicht, was immer wieder einmal – auch vom Autor – empfohlen wird, nämlich statistische Indikatoren nicht rein deskriptiv wirken zu lassen (was meist zu nichts führt), sondern sie in ein System von Forschung und (Politik)Beratung einzubinden, das auf Wirkungsanalysen abstellt (vgl. dazu auch Deutscher Bundestag 2013, S. 284 und Wagner 2013, S. 8 ff.). Dafür nutzen deskriptive Indikatoren-Systeme praktisch Garnichts, sondern es ist notwendig der Forschung möglichst gute empirische Analysen zu ermöglichen. Der in der Politikberatung erfahrene Ökonom Christoph Schmidt schreibt dazu (2018, S. 124): „Die ganzheitliche Wohlfahrtsberichterstattung >jenseits des BIP< zieht ihre potenzielle Stärke daraus, soziale und ökologische Aspekte gleichberechtigt neben die ökonomische Dimension der Nachhaltigkeit zu setzen. Allerdings kann die Berichterstattung über einen fest definierten Indikatorensatz nicht die fachkundige Begutachtung durch Experten und den darauf aufbauenden Diskurs über die betrachteten Zusammenhänge ersetzen. Eine eigenständige Legitimation wird diese Entwicklungslinie daher nur durch die Einrichtung eines regelmäßigen Verfahrens der unabhängigen Begutachtung erreichen können, das ihre Natur als Diskursinstrument unterstreicht. Orientieren könnte sich dieses Verfahren am Beispiel der Jahresgutachten der ‚Wirtschaftsweisen‘, die im jeweils folgenden Jahreswirtschaftsbericht des Bundeswirtschaftsministeriums kommentiert werden. Einer Begutachtung der statistischen Kennzahlen eines Indikatorentableaus durch fachkundige Expertengremien würde dann eine Stellungnahme der Bundesregierung und ein öffentlicher Diskurs folgen. Die Begutachtung eines Indikatorensystems würde daher nicht den Abschluss dieses Diskurses, sondern vielmehr seine fachliche Fundierung darstellen.“ Karl Ulrich Mayer, ein Schüler von Wolfgang Zapf, dem deutschen Pionier der Sozialindikatorenbewegung, bringt die Sache in seiner zusammenfassenden Kommentierung von Schmidt (2018) auf den Punkt: „Schmidt [rät] dazu, Indikatoren und Wohlfahrtsberichte immer von wissenschaftlichen Experten interpretieren zu lassen. Eine Ergänzung bzw. Alternative dazu wäre es, Indikatorensysteme sehr viel expliziter, als dies meistens geschieht, in umfassendere Datenerhebungen und Datenanalysen einzubetten (wenn nicht sogar, sie dadurch zu ersetzen).“ (Mayer 2018, S. 16f).^6^

Interessanterweise will die Ampel-Bundesregierung laut Koalitionsvertrag in diese Richtung gehen und die evidenzgestützte Politikberatung stärken. So soll der Jahres*wirtschafts*bericht zu einem Jahres*wohlstands*bericht ausgebaut werden; und mit einem entsprechenden Spezialkapitel im ersten von der Ampel verantworteten Jahreswirtschaftsbericht wurde in ganz kurzer Zeit auch ein erster Schritt gemacht (BMWK 2022, S. 79 ff.). Und im Koalitionsvertrag (S. 32, Abschnitt „Bürokratieabbau“) heißt es „Wir werden die wissenschaftlichen Beratungsgremien der Bundesregierung nach dem Vorbild der Monopolkommission stärken und deren Unabhängigkeit garantieren. Die Berichte der Sachverständigenräte werden wir nach ihrer Veröffentlichung im Bundestag als eigenständigen Tagesordnungspunkt diskutieren.“

Darüber hinaus wird die Problematik der „Nutzung von Daten und Datenrecht“ im Koalitionsertrag auf Seite 17 wie folgt angesprochen: *„*Die Potenziale von Daten für alle heben wir, indem wir den Aufbau von Dateninfrastrukturen unterstützen und Instrumente wie Datentreuhänder, Datendrehscheiben und Datenspenden gemeinsam mit Wirtschaft, Wissenschaft und Zivilgesellschaft auf den Weg bringen. (…) Ein Dateninstitut soll Datenverfügbarkeit und -standardisierung vorantreiben, Datentreuhändermodelle und Lizenzen etablieren. (…) Mit einem Datengesetz schaffen wir für diese Maßnahmen die notwendigen rechtlichen Grundlagen. Wir fördern Anonymisierungstechniken, schaffen Rechtssicherheit durch Standards und führen die Strafbarkeit rechtswidriger De-Anonymisierung ein. Wir führen einen Rechtsanspruch auf Open Data ein und verbessern die Datenexpertise öffentlicher Stellen.*“*

Soziale Indikatoren könnten also, eingebunden in eine neuartige Diskurs-Kultur, in Zukunft in der deutschen Politik eine größere Rolle spielen. Was aus dem Koalitionsvertrag wird, muss man allerdings – wie bei jedem Koalitionsvertrag – abwarten (vgl. auch Bender und Schmidt 2022).

Was ist die oben angesprochene positive Lektion der Pandemie? Nun, der öffentliche Umgang mit der Pandemie hat gezeigt, dass die breite Öffentlichkeit in der Lage ist statistische Informationen sinnvoll einzuordnen und zu verarbeiten. Nicht alle wollen das tun, aber eine Mehrheit macht das. Dies zeigt über Corona hinaus, dass es keineswegs aussichtslos ist mit Hilfe von Statistik mehr Rationalität in öffentliche Debatten und Auseinandersetzungen zu bringen. Und es dafür noch nicht einmal der Aufbau von „Statistical Literacy“ in der Schule notwendig (der aber nicht falsch, sondern sehr nützlich wäre; vgl. Krämer et al. 2019), sondern schlicht und einfach nur ein ausreichend großer Problemdruck. Der war bei der Pandemie gewissermaßen „von selbst“ gegeben – für andere Bereiche (z. B. im Bereich der Einkommens- und Vermögensverteilung) muss man sich Gedanken machen wie man öffentliches Problembewusstsein schafft. Darüber sollten nicht nur Kommunikationswissenschaftler mehr nachdenken, sondern auch Statistiker. Das tun sie auch wie die Stellungnahme der Deutschen Arbeitsgemeinschaft Statistik im Jahr 2021 zu „Daten und Statistik als Grundlage für Entscheidungen: Eine Diskussion am Beispiel der Corona-Pandemie“ beweist (Behnke et al. 2021). Darin werden ausdrücklich Unsicherheiten von Daten und Entscheidungsmodellen genannt (was die Glaubwürdigkeit der Wissenschaft stärkt) und die Förderung von Statistical Literacy betont (vgl. auch Radermacher 2022, S. 395). Und Berger et al. (2022, S. 388 f.) stellen ausdrücklich „Reporting und Communication“ als wichtige Aufgabe heraus.

## Footnotes

1 Der britische Ökonom Charles Goodhart hat in den 70er-Jahren die These aufgestellt, dass Indikatoren, die zu Zielen zur Steuerung der Geldpolitik gemacht werden, in diesem Moment ihre Aussagekraft verlieren, da sie fortan manipuliert werden. Die Verallgemeinerung dieses Gedankengangs findet man bereits bei Campbell (1976); vgl. auch Rodamar (2018).

2 Interessanterweise beruht die Volkswirtschaftliche Gesamtrechnung in Teilen auf Nowcasts, also Schätzungen bestimmter Aggregate; was in der öffentlichen Diskussion nahezu immer ignoriert wird, aber anhand der vielfachen Revisionen bis zum endgültigen Ergebnis deutlich wird.

3 Der R‑Wert gibt an, ob die Zahl der Neuinfektionen zu einer sinkenden (R < 1), gleichbleibenden (R = 1) oder steigenden Bestandszahl (R > 1) an Infizierten führt. Die Verdopplungszeit gibt bei steigenden Zahlen an, wie lange es dauert, bis der Bestand an Infizierten sich verdoppelt hat.

4 Auf das ebenfalls aufgekommene Problem der mehr oder weniger systematischen Desinformation soll hier nicht näher eingegangen werden (vgl. dazu z. B. Leuker et al. 2022).

5 Ob die empfohlene regierungsamtliche „Einrichtung einer Bundesstelle zur Entwicklung und Implementation von Strategien gegen Falschinformationen“ angesichts vielerlei Verschwörungstheorien eine besonders kluge Empfehlung ist, und eine wissenschaftsgetragene Einrichtung nicht effektiver wäre, soll hier nicht diskutiert werden.

6 Vgl. auch Wagner (2013, S. 8): „Die Wichtigkeit der Einbettung von statistischen Indikatoren in ein geeignetes ‚Berichtssystem‘ wird von der Enquete-Kommission ‚Wachstum, Wohlstand, Lebensqualität‘ ausdrücklich betont (Deutscher Bundestag 2013, S. 284). Danach ist es ‚recht naiv zu glauben, dass neuartige Indikatoren und ihre fundierte Zusammenstellung in einem Indikatorensatz aus sich selbst heraus wirkmächtig werden können. Stattdessen dürften statistische Indikatoren und ihre Zusammenstellung in einem Indikatorensatz nur dann eine große Wahrnehmung im öffentlichen Diskurs erfahren, wenn sie gleichrangig mit dem BIP kommuniziert werden“. Die Frage lautet also: Wie kann ein institutioneller ‚Unter- und Überbau‘ aussehen, der dafür sorgt, dass ein auf die ganzheitliche Erfassung von Wachstum, Wohlstand und Lebensqualität ausgerichteter Indikatorensatz ‚in aller Munde‘ ist? Die Enquete fährt fort: „Nur wenn die Bundesregierung das Tableau politisch ernst nimmt, kann es wirkmächtig werden. Deswegen ist nach Überzeugung der Enquete-Kommission eine (Selbst)Verpflichtung der Bundesregierung, dass sie zu dem durch den Indikatorensatz regelmäßig ausgewiesenen Stand von Wachstum, Wohlstand und Lebensqualität in konsistenter Weise explizit Stellung bezieht, von entscheidender Bedeutung. Diese Stellungnahme müsste über die jährlichen ‚Jahreswirtschaftsberichte‘ hinausgehen und organisatorisch beim Bundeskanzleramt verankert sein.“.
